# Epstein-Barr Virus–Associated Pulmonary Leiomyoma in a Patient With Untreated Human Immunodeficiency Virus Infection

**DOI:** 10.1093/ofid/ofad492

**Published:** 2023-10-03

**Authors:** Shivani Sharma, Joseph Ulicny, Moe Thuzar, Roberto Silva Aguiar, Sarah Sharkey, Frank Zhang, David Chambers, Alexandre E Malek

**Affiliations:** Department of Internal Medicine, Louisiana State University Health, Shreveport, Louisiana, USA; Department of Internal Medicine, Louisiana State University Health, Shreveport, Louisiana, USA; Department of Pathology, Louisiana State University Health, Shreveport, Louisiana, USA; Department of Pathology, Louisiana State University Health, Shreveport, Louisiana, USA; Department of Pulmonary and Critical Care, Louisiana State University Health, Shreveport, Louisiana, USA; Department of Pulmonary and Critical Care, Louisiana State University Health, Shreveport, Louisiana, USA; Department of Pulmonary and Critical Care, Louisiana State University Health, Shreveport, Louisiana, USA; Division of Infectious Disease, Department of Internal Medicine, Louisiana State University Health, Shreveport, Louisiana, USA

**Keywords:** EBV, endobronchial, HIV, leiomyoma, smooth muscle tumor

## Abstract

We report an Epstein-Barr virus–associated smooth muscle tumor in an adult male with AIDS. The patient had multiple lung nodules seen on computed tomography of the chest and an endobronchial lung tumor identified on bronchoscopy. Initiation of antiretroviral therapy slowed the progression of the tumors.

Primary pulmonary tumors of smooth muscle origin comprise <2% of all benign lung tumors [1]. These tumors arise from the smooth muscle cells of the bronchi or bronchiole and can present as parenchymal or endobronchial lesions [2]. Epstein-Barr virus (EBV)–related lung leiomyoma and leiomyosarcoma have been described in case reports in patients with untreated human immunodeficiency virus (HIV) infection [3–5]. The exact mechanism of tumorigenesis is unclear, but it seems to be related to EBV infection and the transformation of the smooth muscle cells [6]. We report a rare case of multicentric pulmonary smooth muscle tumors (SMTs) in an adult male with untreated HIV.

## CASE REPORT

**Figure 1.**
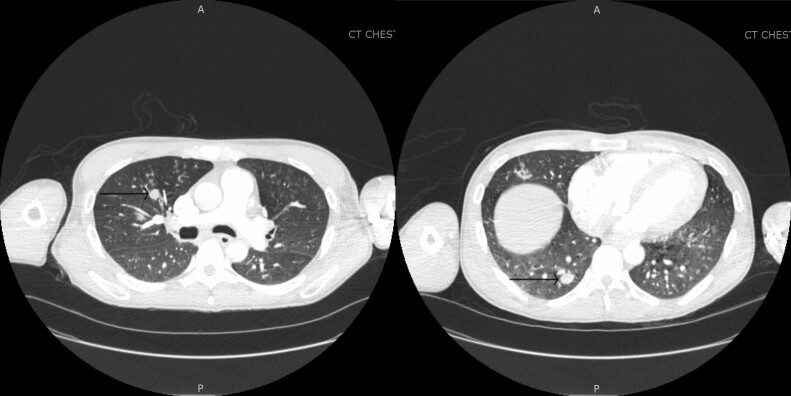
Patient's chest computed tomography (CT) at presentation. Arrows are showing multiple lung nodules of varying sizes.

**Figure 2.**
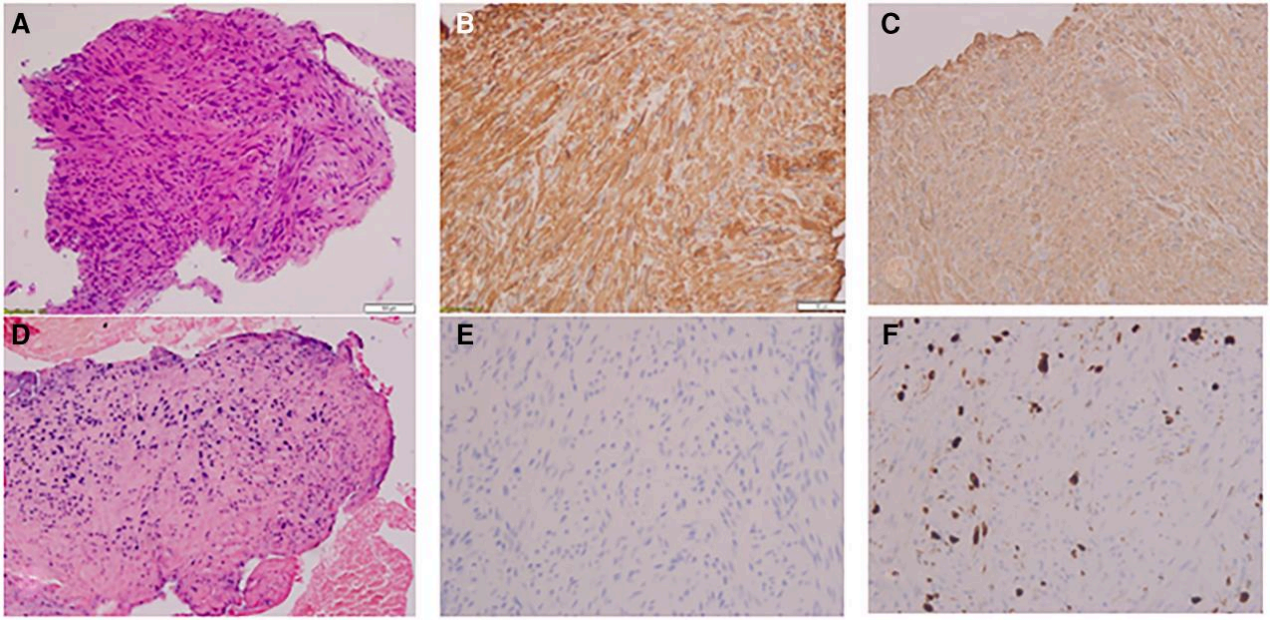
Primary pulmonary leiomyoma. *A*, Hematoxylin and eosin stain: fragment of the tumor showing well-differentiated spindle cells with mature chromatin and rounded edges. Cytoplasm is eosinophilic, and no mitotic figure or necrosis is observed. *A–E*, Immunohistochemical staining was positive for caldesmon (*B*), smooth muscle actin (*C*), and Epstein-Barr encoding region in situ hybridization (*D*) and negative for human herpesvirus 8 (*E*). *F*, Ki-67 is 10%–15%, signifying a low proliferate status and a benign nature.

A 34-year-old man with a medical history of untreated advanced HIV, schizoaffective disorder, and substance use disorder was brought to the emergency department due to reported delusions of grandeur and bizarre behavior. He endorsed fever and productive cough for a few days prior to the presentation. Vital signs showed a temperature of 40°C (104°F), blood pressure of 134/77 mm Hg, heart rate of 106 beats per minute, and peripheral oxygen saturation of 100% on room air. Upon physical examination, he was found to have rhonchi on lung auscultation bilaterally. He did not have any focal neurological deficits. The patient was reportedly first diagnosed with HIV in 2011 but had been lost to follow-up and off of antiretroviral therapy (ART) for the last 5 years. Laboratory investigations revealed a white blood count of 1.20 K cells/μL and a total lymphocyte count of 0.8 K cells/μL; red blood count and platelets were within normal range. HIV RNA level was 279 235 copies/mL, and CD4^+^ T-cell count was 4 cells/μL. The urine drug screen was positive for methamphetamine and marijuana. Computed tomography (CT) of the chest at this presentation revealed worsening of bilateral scattered noncalcified pulmonary nodules of varying size as compared to the CT scan from 6 months prior (Figure 1). A sputum polymerase chain reaction for *Pneumocystis jirovecii* (PJP) was negative. Fungal workup was done for lung nodules, including Fungitell, serum cryptococcal antigen, serum and urine histoplasma antigens, *Blastomyces* antigen and antibody, and *Coccidioides* antibody, which were all unremarkable. CT of the head without contrast and magnetic resonance imaging of the brain without contrast did not show acute intracranial process. In addition, a lumbar puncture was done, and the infectious workup of the cerebrospinal fluid—including gram stain and culture, herpes simplex virus, human herpesvirus 6, varicella zoster virus, cytomegalovirus, *Enterovirus*, and *Cryptococcus*—was negative. The patient received a course of vancomycin and cefepime therapy for healthcare-associated pneumonia. His fever and cough resolved with antibacterial treatment. He was started on trimethoprim-sulfamethoxazole for PJP pneumonia prophylaxis. The patient was also initiated on ART with bictegravir, emtricitabine, and tenofovir alafenamide. The patient underwent robotic-assisted bronchoscopy, which showed a single oval, smooth, and vascular lesion with complete obstruction in the right anterior segment (RB3) of the lung. Bronchoalveolar lavage (BAL) and multiple endobronchial and transbronchial biopsies were taken. Bacterial, fungal, and acid-fast bacilli cultures in BAL were negative. Pathology from the biopsies came back positive for leiomyoma. Immunostaining was positive for caldesmon, smooth muscle actin (SMA), Ki-67 (10%) (Figure 2). Epstein-Barr encoding region (EBER) in situ hybridization was positive in 65% of the nuclei. As EBV-associated leiomyomas are known to involve multiple organs, a CT of the abdomen and pelvis with contrast was done. It showed enlarged retroperitoneal and inguinal lymph nodes. The patient had an excisional biopsy of the inguinal lymph node, which showed a benign reactive lymph node compatible with HIV-related lymphadenopathy. The patient’s delusions resolved and were thought to be related to methamphetamine use. He was discharged on ART with close follow-up with Infectious Diseases and Pulmonology. The patient presented for follow-up at 3 months and was asymptomatic, although he was noted to have high viremia (HIV RNA 40 451 HIV copies/mL, and CD4^+^ T-cell count was 5 cells/μL), suggestive of poor adherence. CT of the chest showed grossly stable multiple solid noncalcified bilateral pulmonary nodules. He was counseled on the importance of adherence to ART. Continued observation of the lung nodules with imaging is planned.

## DISCUSSION

EBV-associated SMTs are seen in young, severely immunocompromised patients, as in our case [7, 8]. Patients with endobronchial lesions can be asymptomatic or present with various symptoms depending on the tumor’s size and location [9, 10]. In previous reports, patients have presented with cough, shortness of breath, wheezing, hemoptysis, fever, and weight loss [3–5, 11]. Our patient with untreated HIV was asymptomatic for 6 months despite having worsening lung nodules. His clinical presentation with fever and cough was most likely attributed to bacterial pneumonia, and his symptoms resolved with antibiotic therapy. Bronchoscopy with biopsy is needed for a definitive diagnosis. Histologically, leiomyoma demonstrates spindle-shaped cells. Immunostaining can be positive for muscle markers like SMA, α-smooth muscle actin, and caldesmon. The presence of EBV within SMT can be proven with positive EBER in situ hybridization, which is the gold standard test for diagnosis. Our patient had positive immunostaining for caldesmon, SMA, and positive EBER.

These tumors are seen in patients with impaired cell-mediated immunity, including patients with untreated HIV, posttransplant-related immunosuppression, or congenital immunodeficiency [12]. In patients with untreated HIV, EBV-SMTs of the brain, spinal cord, liver, lung, and adrenal glands have been described [7]. Multiorgan system involvement is more common as compared to single-organ involvement [7]. Our case is unique as the patient had primary pulmonary involvement and did not exhibit evidence of any other organ involvement on imaging.

EBV-associated endobronchial leiomyoma is primarily described in children with advanced HIV [8]. In a literature review comprising 64 cases of EBV-SMTs in adult patients with advanced HIV, 12 patients had lung involvement [9]. In addition to our case report, we could identify very few case reports of unicentric and multicentric primary endobronchial smooth muscle tumors in people with HIV [3–5]. EBV-SMTs after solid organ transplantation are rare and can develop in the graft or any other organ [13], with the highest incidence after kidney transplant followed by liver, heart, and lung [14]. They are most frequently seen in the liver, followed by respiratory and gastrointestinal tracts [13, 14]. In transplant recipients, posttransplant lymphoproliferative disorder (PTLD) is seen more commonly than EBV-SMTs [15]. The clinical presentation of PTLD after lung transplant depends on the time elapsed from transplantation to the diagnosis of PTLD [16]. Disease occurring within the first year is more likely to present in the thorax, including allograft parenchyma or mediastinal lymph nodes [15]. After the first year of transplant, extrapulmonary presentation, including the gastrointestinal tract, is common [15, 16]. The symptoms of PTLD can be systemic, including fever, fatigue, and sweats, and will also depend on the site involved [17].

Different mechanisms have been proposed for the entry of EBV in the smooth muscle cells. One of them is mediated by the human complement receptor 2 (CD21) [6]. In patients with untreated HIV, increased expression of CD21 receptors has been found in tumor cells from EBV-SMTs [6, 18], whereas in posttransplant EBV-SMTs, CD21 is rarely expressed [19]. Another proposed mechanism of entry is the fusion of EBV-infected lymphocytes with smooth muscle cells [20]. After entering the cell, it is unclear how EBV infection causes neoplastic transformation and clonal proliferation. Studies have shown that EBV-SMTs in both untreated HIV and posttransplant have activated phosphoinositide 3-kinase/mammalian target of rapamycin signaling [21]. In cases of multiple nodules or multiorgan involvement, it is hypothesized that multiple lesions do not represent metastases but rather independent malignant transformation events, based on clonality studies of the virus [6, 22–24].

The treatment of endobronchial lesions focuses on local control. Patients with endobronchial leiomyoma were managed with bronchoscopic tumor excision and close follow-up [3, 4, 11]. Increased development of EBV-SMTs in patients with impaired cellular immunity shows that T-cell immunity has a critical role in the suppression of neoplastic transformation driven by EBV. In a few case reports, initiation of ART alone has been associated with slowed tumor progression [25]. Relevant to this presentation, surgical resection in combination with ART has been shown to produce remission [26, 27]. Our patient had stable lesions 3 months after diagnosis, but he was not fully adherent to ART, as evidenced by persistent viremia and low CD4. Notably, in a review of 64 patients of EBV-SMTs by Purgina et al, the leading cause of mortality in patients with leiomyoma was due to complications of immunosuppression, such as opportunistic infection, and not directly related to the EBV-SMTs [9].

## CONCLUSIONS

EBV-associated pulmonary leiomyoma should be considered in the differential diagnosis of patients with untreated HIV presenting with lung nodules or endobronchial lesions. Healthcare practitioners should maintain a high clinical suspicion of this rare condition as it is of utmost importance to request EBV staining of the tissue sample for proper diagnosis. Furthermore, increased awareness of this disease process is crucial to prevent empirical treatment for atypical pneumonia with unnecessary, potentially toxic antibiotic and antifungal regimens. The true incidence of EBV-associated smooth muscle tumors in patients with untreated HIV remains unknown. Further studies are needed to determine the optimal management and ensure successful outcomes.

## Notes

***Patient consent.*** Patient consent not required as patient's pictures have not been used and all the patient information has been anonymised.
